# Approach selection of radiofrequency catheter ablation for ventricular arrhythmias originating from the left ventricular summit: potential relevance of Pseudo Delta wave, Intrinsicoid deflection time, maximal deflection index

**DOI:** 10.1186/s12872-017-0575-5

**Published:** 2017-05-30

**Authors:** Cheng Zheng, Jin Li, Jia Li, De-Pu Zhou, Xiao-Wei Li, Shu-Jie Wu, Jia-Feng Lin

**Affiliations:** Department of Cardiology, Second Affiliated Hospital of Wenzhou Medical University, 109 Xueyuan Road, Wenzhou, Zhejiang 325000 China

**Keywords:** Catheter ablation, Radiofrequency current, Ventricular arrhythmia, Cardiac electrophysiology, Great cardiac vein, Left ventricular summit

## Abstract

**Background:**

Ventricular arrhythmias (VAs) originating from the left ventricular summit is a challenge for radiofrequency catheter ablation (RFCA). The present study aimed to investigate the appropriate RFCA strategy for VAs originating from the left ventricular summit.

**Methods:**

Forty-five consecutive patients with VAs arising from the left ventricular summit were successfully ablated at our cardiac electrophysiology center and reviewed in the study.

**Results:**

Thirty-two cases of VAs were eliminated in the left ventricular endocardium by retrograde transaortic (*n* = 22, 22/45, 48.9%) or antegrade transseptal (*n* = 10, 10/45, 22.2%) approaches, the other 13 cases were eliminated in the left ventricular epicardium by distal great cardiac vein (DGCV) approach (*n* = 13, 13/45, 28.9%). Though these VAs were similar in electrocardiographic (ECG) morphology, the pseudo delta waves (PDW), intrinsicoid deflection time (IDT), maximal deflection index (MDI) differed among them, PDW >53 ms, IDT > 74 ms, MDI > 0.45 strongly indicated that ablating left ventricular summit VAs by DGCV approach. During mean follow-up of 19.5 ± 13.2 (range, 3-60) months, 2 (4.4%) patients experienced VAs recurrence.

**Conclusion:**

This retrospective study showed that VAs of left ventricular summit origin can be effectively cured with RFCA. For these VAs, prolonged PdW, IDT, MDI indicating RFCA by DGCV approach can be attempted firstly.

## Background

Idiopathic ventricular arrhythmias (IVAs), which include premature ventricular contractions (PVCs) and ventricular tachycardias (VTs), are the most common arrhythmias observed in patients without structural heart disease. Though most of them can be effectively and safely treated with radiofrequency catheter ablation (RFCA) [1–6], VAs arising from an area in the anterosuperior left ventricle outflow tract (LVOT), which is epicardially named Left ventricular summit, occupying the most superior left ventricle (LV) portion, remains a challenge for RFCA [7]. Previous studies reported that left ventricular summit VAs can be ablated by retrograde transaortic approach, antegrade transseptal approach, DGCV approach and even via suboxiphoid transpericardial approach [8–11]. The present retrospective study aimed to explore the most appropriate RFCA strategies for VAs originating from the left ventricular summit.

## Methods

### Study population

From July 2006 to July 2014, 1199 consecutive patients (497 male; mean age 48.2 ± 17.2 [7-86] years), who were proven not to have structural heart diseases by routine biochemistry examination, CT scan, echocardiography, and even coronary angiography and cardiac magnetic resonance in some special cases, received RFCA for VAs at our cardiac electrophysiology center. Of these, 45 patients (3.75%; 24 males; mean age, 56.6 ± 16.4 [17-80] years) with ECG suggestive of VAs arising from the left ventricular summit, which was further confirmed by the effective target on fluoroscopy and three-dimension mapping system (Carto 3 or Ensite-NavX system), were enrolled in the present study. Transesophageal echocardiography was only examined on older patients to exclude thrombus in left atrial appendage because of the potential risk of asymptomatic atrial fibrillation. All patients signed the informed consent form and agreed to the electrophysiological examination and radiofrequency ablation treatment. All these treatments patients received were considered standard care for their conditions.

### ECG measurements

Twelve-lead ECGs of the clinical arrhythmias were obtained and analyzed before RFCA. No patients had T inversion, pathological Q wave in precordial leads or epsilon wave. ECG pattern analysis focused on (1)QRS morphology of the clinical VAs in all 12 leads; (2) the axis deviation; (3) Transitional zone index in precordial leads (defined as transition zone score of VAs minus transition zone score of sinus beat); (4)QRS duration; (5)The pseudo delta wave time (PdW, the interval from the beginning of the QRS complex to the earliest fast deflection in any precordial lead) [11]; (6) The intrinsicoid deflection time (IDT, the interval from the beginning of the QRS complex to the peak of the R wave in V2) [11]; (7)The maximum deflection index (MDI, IDT divided by the QRS duration) [11]; (8) QS wave ratio of aVL/aVR. ECG of each patient was measured by 2 cardiologists unknown the site of origin; any discrepancy was judged by a third cardiologist. Capital letters (Q,R,S) represented waves of relatively high amplitude(>0.5 mV), while lowercase letters (q,r,s) represented waves of relatively low amplitude(<0.5 mV).

### Electrophysiological study and radiofrequency ablation

Electrophysiological study was performed after discontinuation of anti-arrhythmic drugs for at least five half-lives. Catheters were delivered to the distal coronary sinus (CS) via subclavian or femoral veins and to the right ventricle via femoral veins. 12-lead ECG and intracardiac electrogram were monitored and recorded on a multichannel oscilloscopic recorder. If the clinical arrhythmias didn’t appear at the beginning of the electrophysiologic study, induction of VAs was attempted by programmed electrical stimulation from the right ventricular apex, delivering a maximum of three extrastimulis with basic drive cycle lengths of 600, 500, and 430 ms. If VAs was still not provoked at baseline, intravenous isoproterenol (2–4 μg/min) was administered. In all case, activation mapping and pace mapping were both performed to identify the VA origin site. The activation time was measured during VA from the onset of the ventricular activation of the distal bipole of the mapping catheter to the earliest onset of the QRS complex in any of the 12 ECG leads. Pace mapping was performed during sinus rhythm at a pacing cycle length of 500 ms with stimulus amplitude of 1 mA greater than the late-diastolic threshold, the pace match was assessed by whether the paced QRS morphology match the clinical VAs in the 12-lead ECG (a perfect pace map was defined as 12 leads complete match, an excellent pace map referred to more than 10 leads match). When a site was mapped with a local ventricular activation preceding the QRS onset by more than 20 ms and pacing from the site produced an excellent match to the QRS complex of the clinical VAs, catheter ablation was attempted.

For these VAs, complete LVOT mapping and ablation by retrograde transaortic approach and antegrade trans-DGCV approach was performed in every patient. When endocardial catheter ablation was unsuccessful with a transaortic approach or a much earlier local ventricular activation was mapped in the DGCV than at any other endocardial site, epicardial catheter ablation by trans-DGCV approach was attempted. If both transaortic and trans-DGCV RFCA didn’t abolish the VAs, mapping and ablation below aortic root via a transseptal approach was finally attempted. Additional LVOT mapping by antegrade transseptal approach was performed for the reason that only by retrograde transaortic approach, the anatomic area just below the left coronary cusp (LCC), which was the endocardial side of left ventricular summit, was sometimes insufficiently mapped. Antegrade transseptal approach was achieved under the guidance of fluoroscopy with transseptal puncture through the anteroinferior fossa ovalis, slightly posterior to the mapping catheter placed in the noncoronary sinus cusp. After transseptal puncture, the reversed S curved ablation catheters was delivered to the area just below the LCC in the left ventricular outflow tract. With the assistance from an Agllis NxT sheath, the ablation catheters were manipulated with ease in this area. During the whole procedure, activated clotting times was monitored and maintained between 250 and 300 s by unfractionated heparin to prevent thrombus.

If the overall site of earliest ventricular activation was found within the DGCV or below the LCC, coronary angigraphy was performed to assess the distance between the tip of ablation catheter and left coronary arteries and their ostia, identify the exact anatomic location and avoid the inadvertent damage to coronary artery.

Once a target site was located, RF energy was released by the irrigated-tip catheter in a temperature-controlled mode with a target temperature of 43 °C, maximum power output of 35 W, and flow rate of 17 ml/min, to achieve a impedance decline of 10-15Ω. If ablation in DCGV was required, the irrigated-tip catheter mode was switched to maximum power output of 25 W and flow rate 30 ml/min. During the catheter ablation within DGCV or below LCC, left coronary angiography was repeated every 15 s to evaluate the distance of the ablation catheter relative to the left coronary arteries and their ostia, the energy delivery should be immediately stopped once the catheter slightly dislodged. Energy delivery was forbidden within 5 mm of a coronary artery. Coronary angiography was routinely performed after successful ablation to assess coronary blood supply. During the first 10 s of the energy release on the target site(20 s for antegrade transseptal approach), if the frequency of VT or PVCs accelerated or decelerated, the energy was applied for another 60 s, if not, the energy application was stopped, and another target site was searched for. Programmed electrical stimulation and isoproterenol infusion were both performed before withdraw of all catheters and sheaths. The RFCA endpoint was defined as absence of spontaneous or inducible VT or PVCs.

### Post ablation follow-up

After RFCA, all patients underwent 72-h ECG monitoring deprived of any antiarrhythmic drugs. After discharge, all patients were followed up at our outpatient department. To assess long-term outcome, 24-h Holter monitoring and echocardiography were carried out at 1, 3, 6 and months later. Whenever the patients had symptoms suggesting recurrence of PVCs/IVT, ECG and 24-h Holter were performed immediately. Successful catheter ablation was defined as no recurrence of VAs during follow-up.

### Statistical analysis

All the authors did not have access to identifying information during or after data collection. Continuous data were manifested as mean ± standard deviation (X ± *s*), the analysis of variance (ANOVA) and the student’s t-test were applied to compare the continuous data, while categorical data were manifested as number and percentage, the X^2^-test or Fisher method were applied to compare the categorical data. *P* < 0.05 indicated a significant difference. A Receiver operating characteristic (ROC) curve analysis was used to test the predictive value of PdW, IDT, MDI. A two-tailed value of *P* < 0.05 indicated a statistical significance.

## Results

### Study population

The 45 studied patients were divided into 3 groups based on the ablation approaches finally adopted, by which the VAs was completely eliminated in left ventricular summit: (1) Retrograde transaortic group:22 VAs were ablated by energy delivery retrogradely through the aortic root via a catheter inversion technique in the endocardial left ventricular summit; (2) Antegrade transseptal group:10 VAs were ablated by energy delivery in endocardial left ventricular summit through the atrial septum puncture; (3)Trans-DGCV group:13 VAs were ablated by energy delivery in DGCV. Clinical characteristics of the three groups were displayed in Table 1.

**Table 1:** Baseline characteristics of patients in the transaortic group, transseptal group and trans-DGCV group

Group	Male gender	Age, years	Disease duration, years	PVC count (number/24 h)	Hypertension	Diabetes	LV enlargement	NSVT	PVCs
Transaortic (*n* = 22)	11 (50.0)	55.5 ± 18.8	3.06 ± 2.37	26129 ± 10200	9 (40.9)	1 (4)	8 (36.4)	5	17 (77.3)
Transseptal (*n* = 10)	6 (60.0)	59.7 ± 15.8	2.80 ± 2.24	25025 ± 10078	5 (50.0)	/	3 (30.0)	3	7 (70.0)
Trans-DGCV (*n* = 13)	7 (53.8)	56.1 ± 13.7	2.79 ± 2.35	29543 ± 14197	5 (38.5)	/	4 (30.8)	4	9 (69.2)

Data are presented as mean + SD or n (%). *NSVT* Non sustained ventricular tachycardia

### Results of electrophysiological study, mapping and ablation

In 45 patients, frequent PVCs or nonsustained VTs occurred spontaneously during the procedure, programmed electrical stimulation and intravenous isoproterenol were not applied to provoke the clinical arrhythmias at the beginning of electrophysiological study, but were performed at the ending of RFCA to confirm the absence of the spontaneous and induced ventricular arrhythmias. The target sites of VAs arising from left ventricular endocardial or epicardial summit were confirmed by electrophysiological study, fluoroscopy, coronary angiography and three-dimension electroanatomic mapping. For all the 45 VAs, 22 were successfully ablated by energy delivery in LV endocardial summit by retrograde transaortic approach via catheter tip inversion technique, on bipolar recording, activation mapping showed that earliest ventricular activation of target site preceded the QRS onset by 33.95 ± 5.43 ms during clinical VAs, pace mapping demonstrated excellent match of the paced QRS morphology to the clinical VAs (10.94 ± 1.87 leads concordance), mean time of energy delivery to clinical VA disappearance was 7.4 ± 3.4 s, the VAs were successfully abolished by a mean of 2.58 ± 1.59 radiofrequency applications. 13 VAs were eliminated by trans-DGCV approach, activation mapping showed the earliest ventricular activation of target site preceded the QRS onset by 35.54 ± 6.44 ms during clinical VA, pace mapping at the site of earliest ventricular activation presented excellent match of the paced QRS morphology to the clinical VA (11.5 ± 0.7 leads concordance), mean time of energy delivery to clinical VA disappearance was 7.5 ± 3.1 s, the VAs were successfully abolished by a mean of 2.08 ± 1.23 radiofrequency applications. For the residual 10 VAs refractory to ablation by transaortic approach and trans-DGCV approach, they were finally ablated by antegrade transseptal approach, bipolar recording showed the earliest ventricular activation of target site preceded the QRS onset by 28.23 ± 2.62 ms during clinical VAs, pace mapping at the site of earliest ventricular activation, demonstrating poor match of the paced QRS morphology to the clinical VA (6.67 ± 4.56 leads concordance), mean time of energy delivery to clinical VA disappearance was 10.7 ± 5.2 s, the VAs were successfully abolished with a mean of 2.94 ± 3.28 radiofrequency applications. The total energy application time, procedure time, X-ray exposure time was longest in transseptal approach, for it was the approach finally applied (energy application time: Transseptal 366.90 ± 78.40 ms vs. Trans-DGCV 289.40 ± 58.40 ms and Transaortic 138.90 ± 32.40 ms, *p* < 0.05; procedure time: Transseptal 85.68 ± 21.94 min vs. Trans-DGCV 70.23 ± 22.95 min and Transaortic70.61 ± 21.85 min, *p* < 0.05; X-ray exposure time: Transseptal 10.19 ± 3.94 min vs. Trans-DGCV 9.41 ± 3.92 min and Transaortic 9.08 ± 3.36 min vs. *p* < 0.05, respectively). Of note, the VAs eliminated by transaortic and trans-DGCV approach usually disappeared in 10s of energy delivery on target sites, while most VAs by transseptal approach needed more than 10s to achieve successful ablation. Radiofrequency ablation procedure of these 3 groups are shown in Table 2.

**Table 2:** Mapping and ablation of VAs in the transaortic group, transseptal group and trans-DGCV group

Group	Time of earliest ventricular activation preceding QRS onset, ms	Number of similar lead counts between spontaneous PVCs and Pace	RF lesions prior to success	Target site discharge time for PVCs/VT disappearance, s	Ablation time, s	Procedure time, min	X-ray exposure time, min
TA	33.95 ± 5.43^a^	10.94 ± 1.87^†^	2.58 ± 1.59^a^	7.4 ± 3.4 ^a^	139.9 ± 32.4^*^	70.23 ± 22.95^a^	9.41 ± 3.92^a^
TS	28.23 ± 2.26	6.67 ± 4.56	5.94 ± 3.28	10.7 ± 5.2	366.9 ± 78.4	85.68 ± 21.94	19.08 ± 3.36
TGCV	35.54 ± 6.44	11.5 ± 0.7	2.08 ± 1.23^a^	7.5 ± 3.1^a^	289.4 ± 58.4^‡^	70.61 ± 21.85^a^	10.19 ± 3.94^a^

Data are presented as mean + SD or n (%). Annotation: TA = Transaortic group, TS = Transseptal group, *TGCV* Trans-DGCV group. Compared with TS, ^a^ *P* < 0.01, ^†^ *P* < 0.05; and compared with TGCV, ^‡^ *P* < 0.01

### Electrocardiographic characteristics

For VAs arising from left ventricular summit, QRS morphology was inferior axis and high R waves in leads II, III, aVF and V_4-6_, QS in leads aVL, aVR, rs(S) or Rs in lead I, and R or Rs in leads V_1-3_, no S wave on V4-V6 leads was seen in any patient. All patients presented with precordial transition zone ≤V_1_ and transitional zone index <0. The 12-lead ECG of the 45 patients are summarized in Tables 3 and 4. PdW, QRS duration, IDT, and MDI of VAs were measured to compare the electrocardiographic characteristics among the three groups (see Table 5). Although the three groups present similar electrocardiographic characteristics, compared with transaortic-LV summit and the transseptal-LV summit group, the trans-DGCV LV summit group showed wider PdW, IDT, and higher MDI. (PdW Trans-DGCV62.46 ms ± 3.84 ms vs. Transaortic 44.59 ± 6.67 ms and Transseptal 49.69 ± 9.20 ms, *p* < 0.05; IDT Trans-DGCV 83.00 ± 6.06 ms vs. Transaortic 62.09 ± 9.45 ms and Transseptal 71.10 ± 12.37 ms, *p* < 0.05; MDI Trans-DGCV 0.55 ± 0.08 vs. Transaortic 0.39 ± 0.08 and Transseptal 0.48 ± 0.06, *p* < 0.05, Table 2).

**Table 3:** Electrocardiographic characteristics of VAs in the 45 patients

Number	group	QRS axis	QRS morphology			Transition	PdW	IDT	MDI	aVL/aVR
			I	V_1_	V_4_-V_6_	Zone	ms	Ms		
Case 1	TA	Inferior	rS	R	R	<V_1_	55	61	0.30	1.2
Case 2	TA	Inferior	Rs	R	R	<V_1_	56	71	0.38	0.8
Case 3	TA	Inferior	rs	R	R	<V_1_	39	52	0.42	1.1
Case 4	TA	Inferior	rs	R	R	<V_1_	50	60	0.35	1.0
Case 5	TA	Inferior	Rs	R	R	<V_1_	42	59	0.29	0.9
Case 6	TA	Inferior	rs	R	R	<V_1_	35	79	0.49	1.0
Case 7	TA	Inferior	rS	R	R	<V_1_	46	55	0.37	1.4
Case 8	TA	Inferior	rS	R	R	<V_1_	32	43	0.50	1.3
Case 9	TA	Inferior	Rs	Rs	R	<V_1_	51	80	0.44	0.8
Case 10	TA	Inferior	rs	R	R	<V_1_	44	55	0.33	1.2
Case 11	TA	Inferior	rs	R	R	<V_1_	48	64	0.50	1.0
Case 12	TA	Inferior	Rs	R	R	<V_1_	50	65	0.38	1.1
Case 13	TA	Inferior	rS	R	R	<V_1_	51	63	0.48	1.5
Case 14	TA	Inferior	rS	R	R	<V_1_	33	54	0.35	1.4
Case 15	TA	Inferior	rS	R	R	<V_1_	42	73	0.49	1.2
Case 16	TA	Inferior	Rs	R	R	<V_1_	50	67	0.46	1.3
Case 17	TA	Inferior	rS	R	R	<V_1_	44	49	0.29	1.3
Case 18	TA	Inferior	rs	R	R	<V_1_	46	62	0.45	1.2
Case 19	TA	Inferior	rs	r	R	<V_1_	36	64	0.41	0.9
Case 20	TA	Inferior	rs	R	R	<V_1_	43	75	0.34	1.3
Case 21	TA	Inferior	rs	Rs	R	<V_1_	45	57	0.28	1.0
Case 22	TA	Inferior	rs	R	R	<V_1_	43	58	0.28	1.1
Case 23	TS	Inferior	rs	R	R	<V_1_	67	86	0.56	1.2
Case 24	TS	Inferior	rS	Rs	R	<V_1_	55	70	0.45	1.0
Case 25	TS	Inferior	rS	R	Rs	<V_1_	58	77	0.51	1.8
Case 26	TS	Inferior	rS	Rs	R	<V_1_	53	60	0.41	1.1
Case 27	TS	Inferior	rS	R	R	<V_1_	52	64	0.51	1.
Case 28	TS	Inferior	rS	R	R	<V_1_	69	85	0.55	1
Case 29	TS	Inferior	rS	Rs	R	<V_1_	59	89	0.53	0.9
Case 30	TS	Inferior	R	rsR	R	V_1_ ~ V_2_	42	65	0.43	1.2
Case 31	TS	Inferior	r	rs	R	V_1_	48	57	0.45	1.2
Case 32	TS	Inferior	rs	qR	Rs	<V_1_	42	58	0.40	1.3
Case 33	TGCV	Inferior	rS	R	R	<V_1_	66	75	0.46	1.0
Case 34	|TGCV	Inferior	rS	R	R	<V_1_	59	76	0.51	1.3
Case 35	TGCV	Inferior	rS	R	R	<V_1_	65	87	0.60	1.4
Case 36	TGCV	Inferior	rS	R	Rs	<V_1_	61	76	0.55	1.2
Case 37	TGCV	Inferior	rS	R	R	<V_1_	66	85	0.54	1.5
Case 38	TGCV	Inferior	rS	R	R	<V_1_	65	91	0.63	1.7
Case 39	TGCV	Inferior	rS	R	R	<V_1_	67	82	0.64	1.3
Case 40	TGCV	Inferior	rS	R	R	<V_1_	65	81	0.46	1.1
Case 41	TGCV	Inferior	rS	R	R	<V_1_	55	93	0.62	1.2
Case 42	TGCV	Inferior	rS	R	R	<V_1_	63	80	0.47	1.5
Case 43	TGCV	Inferior	rS	R	R	<V_1_	62	87	0.56	1.3
Case 44	TGCV	Inferior	rS	R	R	<V_1_	56	75	0.47	1.3
Case 45	TGCV	Inferior	rS	Rs	R	<V1	62	85	0.64	1.2

Annotation: *TA* Transaortic group, *TS* Transseptal group, *TGCV* Trans-DGCV group

**Table 4:** Electrocardiographic characteristics of VAs among the three approach groups

Group	12-lead surface ECG					Transition	Transitional zone index
	I	II/III/aVF	V_1_	V_2_-V_3_	V_4_-V_6_	Zone	<0, n (%)
TA	Rs, rS or rs	R	R or Rs or r	R or Rs	R	<V_1_	22 (100)
TS	rS or rs, r	R	R or Rs or qR or rsR or rs	R or Rs	R	≤V_1_	10 (100)
TGCV	rS	R	R or Rs	R or Rs	R	<V^1^	13 (100)

*TA* Transaortic group, *TS* Transseptal group, *TGCV* Trans-DGCV group

**Table 5:** Comparison of electrocardiographic indices of VAs among the three approach groups

Group	PdW, ms	IDT, ms	QRS, ms	MDI
TA	44.59 ± 6.67	62.09 ± 9.45^*^	133.32 ± 9.07	0.39 ± 0.08^*^
TS	49.69 ± 9.20	71.10 ± 12.36	142.43 ± 15.04	0.48 ± 0.06
TGCV	62.46 ± 3.84*	83.00 ± 6.06^*^	150.52 ± 12.33	0.55 ± 0.08^*^

Annotation: *TA* Transaortic group, *TS* Transseptal group, *TGCV* Trans-DGCV group

Compared with TS, *P<0.05

**Fig. 1:**
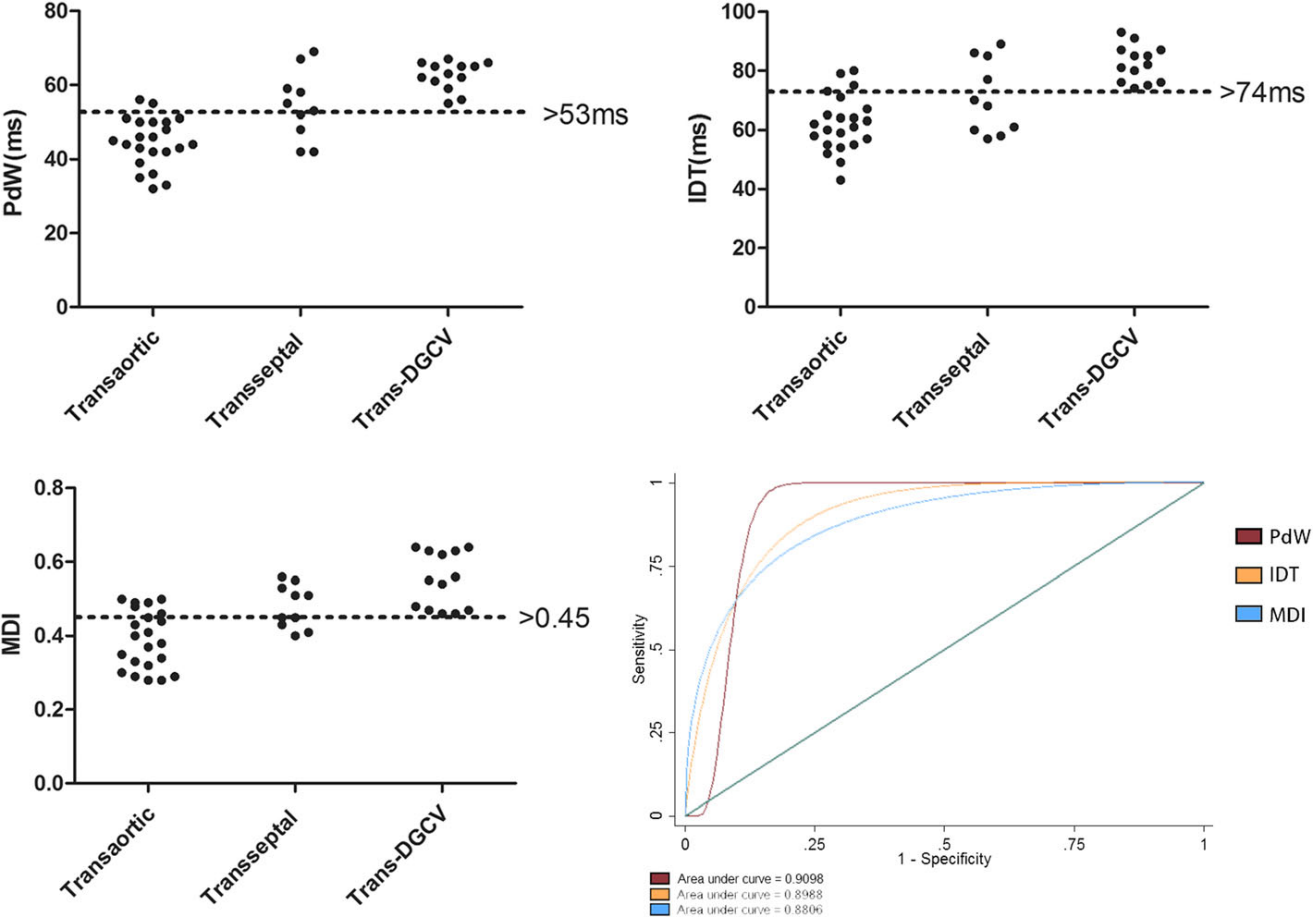
Distribution of PdW time and precordial IDT and MDI values of VAs from transaortic group, transseptal group and trans-DGCV group. PdW > 53 ms, IDT > 74 ms, MDI > 0.45 suggested ablation by the trans-DGCV approach. Receiver Operating characterisitcs (ROC) curve showed that PdW, IDT, MDI of VAs had a good ability to differentiate trans-DGCV approach and non-trans-DGCV approach (transaortic and transseptal approach) with area under curve (AUC) of 0.9098, 0.8988, 0.8806 respectively

**Fig. 2:**
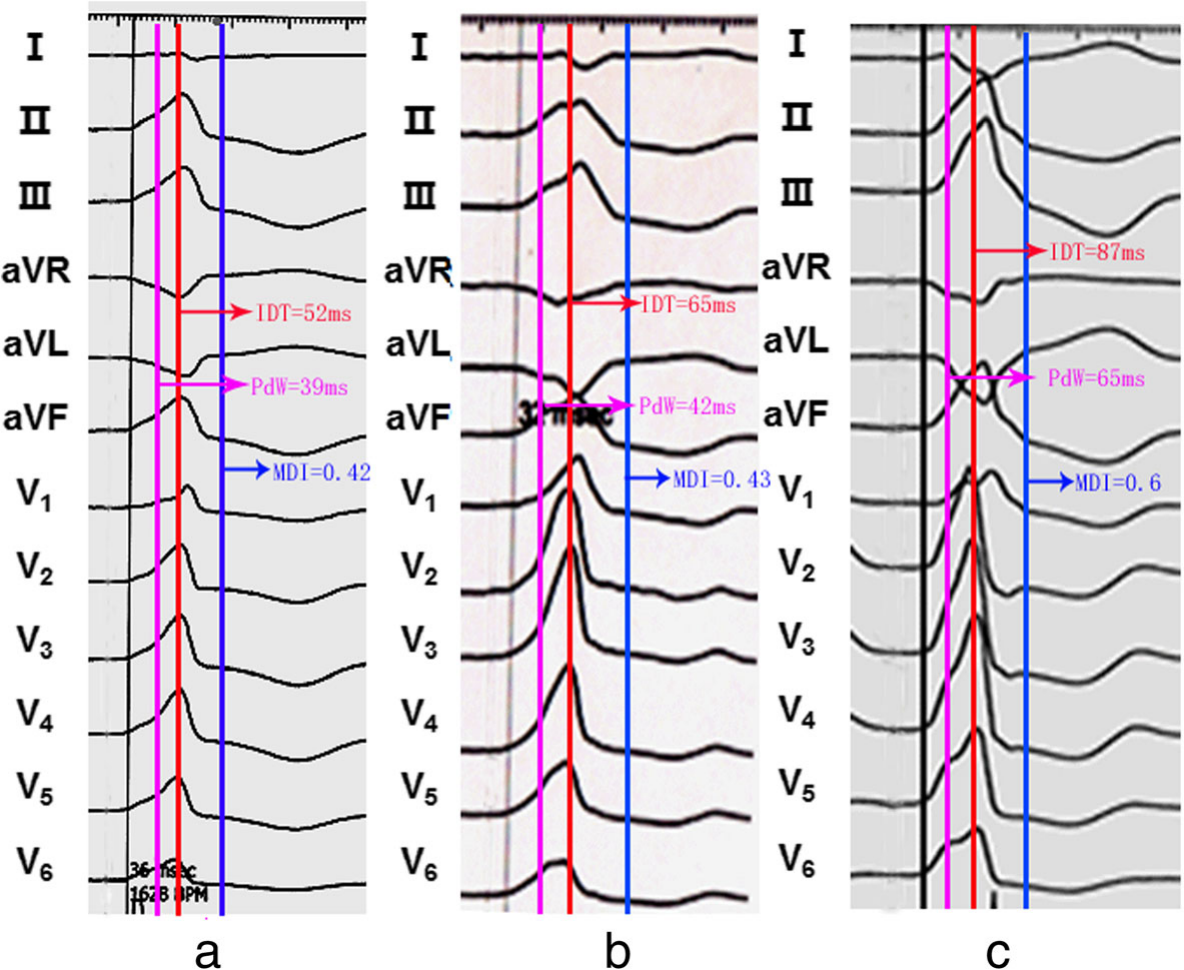
Example of intervals measured in VAs from transaortic group, transseptal group and trans-DGCV group. **a**.Transaortic group **b**.Transseptal group **c**. Trans-DGCV group

**Fig. 3:**
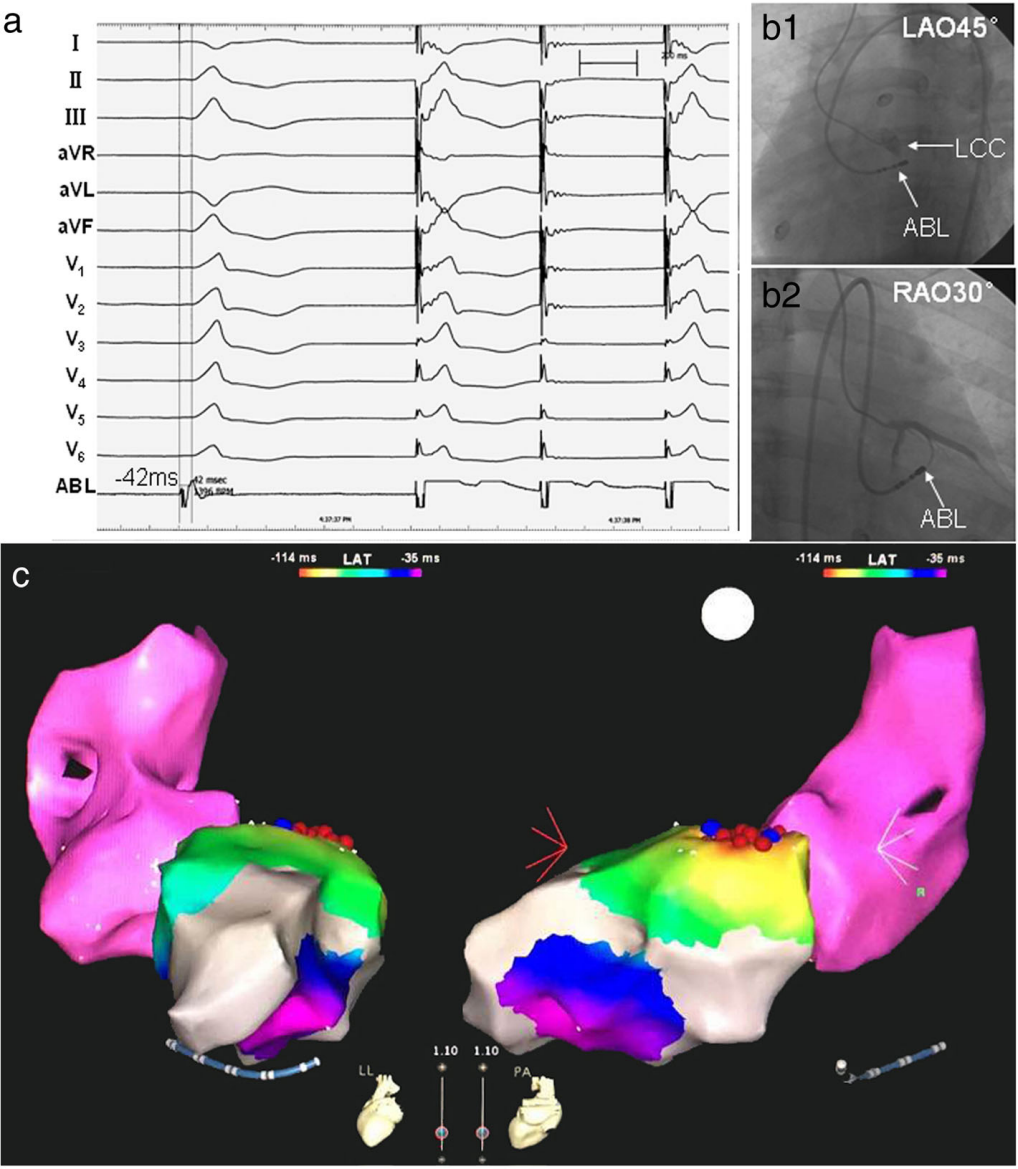
PVCs originating from the *left* ventricular endocardial summit were ablated by a retrograde transaortic approach. **A**. PVCs showed the morphology of complete *right* bundle branch block in precordial leads, with monophasic R wave in leads II, III, aVF, QS in leads aVL and aVR, and rS in lead I. Activation mapping showed that during the sinus rhythm,the earliest ventricular activation of PVCs was recorded in *left* ventricular endocardial summit and preceded the QRS onset by 42 ms; pacing the site of the earliest ventricular activation resulted in a perfect match to spontaneous PVCs. **B.** Fluoroscopy of target site. **C.** Three-dimension mapping of target site. The irrigated-tip ablation catheter was located in the *left* ventricular endocardial summit, and RF current was delivered with a target temperature of 43 °C and maximum power output of 35 W, flow rate 17 ml/min. The PVCs disappeared after energy application for 3 s, and additional RF current then was applied for another 60s. There was no recurrence during 3.5 years follow-up. Annotation: LAO = *left* anterior oblique; RAO = *Right* anterior oblique; ABL = ablation catheter

**Fig. 4:**
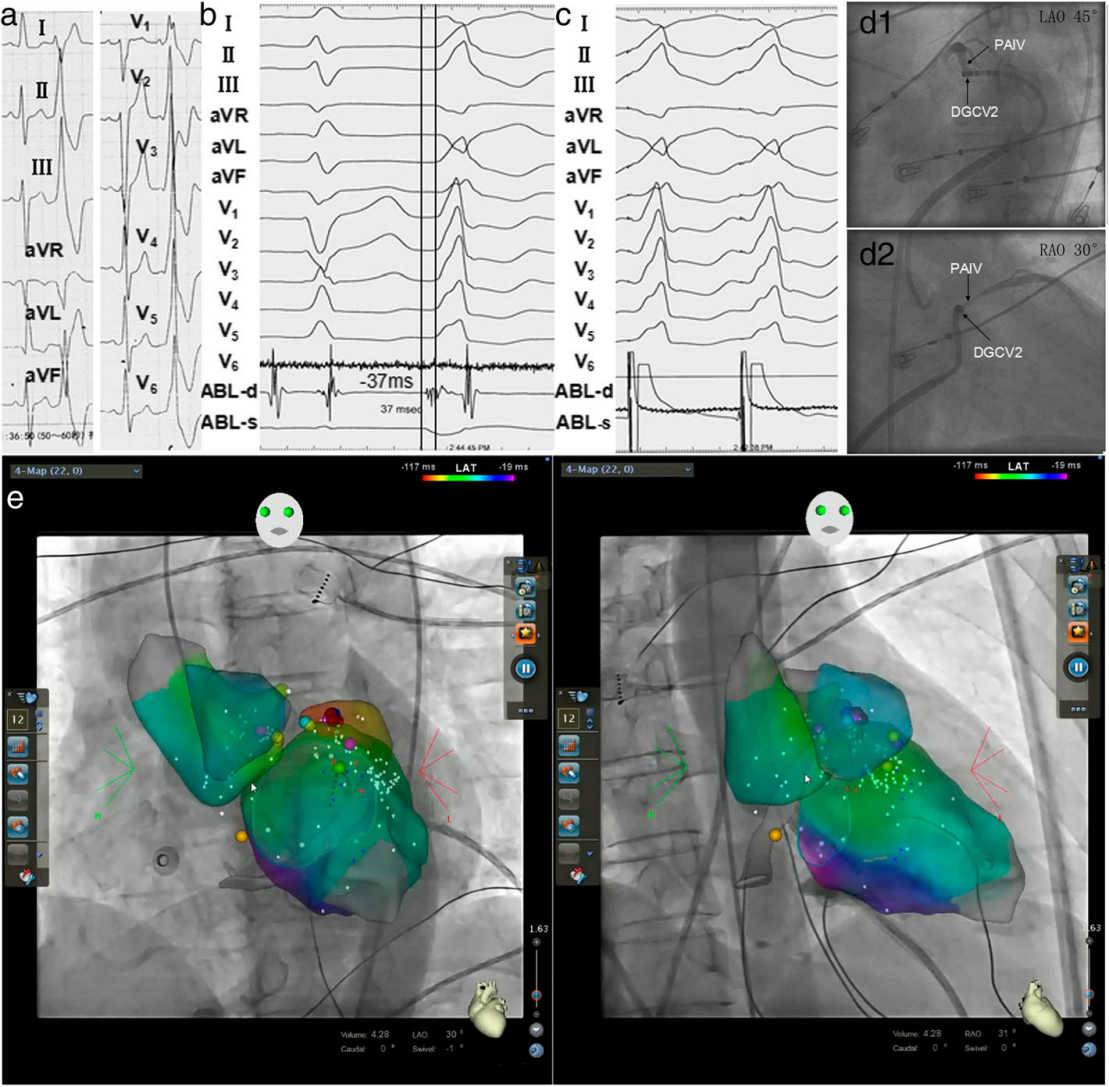
PVCs originating from* left* ventricular epicardial summit were ablated by an antegrade DCGV approach. **a**. PVCs showed the morphology of complete right bundle branch block, with precordial transition zone before V1, monophasic R in leads II, III, aVF and V_3-6_, R_III_ > R_II_, QS in leads aVL and aVR, QS_aVL_ > QS_aVR_, rS in lead I. **b.** The irrigated-tip ablation catheter was delivered to the DCGV via the coronary sinus for mapping and ablation, the earliest ventricular activation of PVCs were recorded in the left ventricular epicardial summit preceding the QRS wave onset by 37 ms. **c.** Pacing the site of the earliest ventricular activation yielded a perfect match to spontaneous PVCs. **d.** Fluoroscopy of target site. **e**. Three-dimension mapping of target site. The ablation catheter was located in the *left* ventricular epicardial summit, and irrigated RF current was delivered with a target temperature of 43 °C, maximum power output of 25 W, and flow rate of 30 ml/min. The PVCs disappeared after energy delivery for 6 s; additional RF current then was applied for another 60s. There was no recurrence during 0.5 years follow-up

**Fig. 5:**
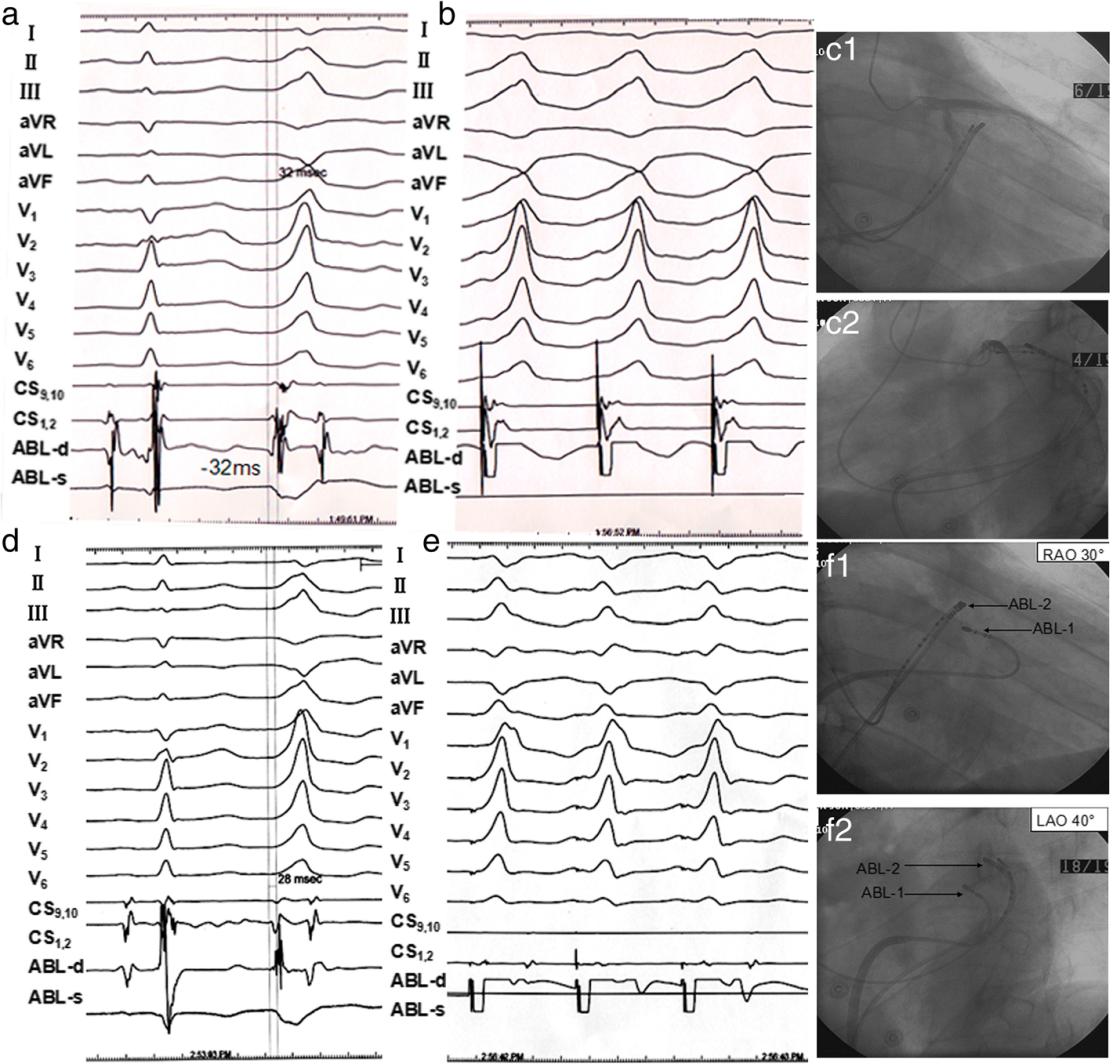
PVCs originating from the *left* ventricular summit were ablated by antegrade transseptal approach. **a**. PVCs showed the morphology of complete right bundle branch block in precordial leads with precordial transition zone before lead V_1_, monophasic R in leads II, III, aVF and V_3-6_, R_III_ > R_II_, QS in leads aVL and aVR, QS_aVL_ > QS_aVR_, rS in lead I. A retrograde transaortic approach was firstly attempted, but failed. The irrigated-tip ablation catheter was then delivered to DCGV via coronary sinus for mapping and ablation. During sinus rhythm, the earliest ventricular activation of PVCs was recorded within DGCV of the left ventricular epicardial summit and preceded QRS wave onset by 32 ms. **b.** Pacing the site of the earliest ventricular activation within DGCV resulted in a perfect match to spontaneous PVCs in 12 leads. **c.** The ablation catheter was located in DCGV, and coronary angiography showed that the distance between the tip of ablation catheter and left coronary artery was more than 5 mm. Irrigated RF current was delivered with a target temperature of 43 °C, maximum power output of 25 W, and flow rate of 30 ml/min. After several times of ineffective RFCA in this area, the trans-DGCV approach was abandoned. **d.** The transseptal approach was finally attempted, by this approach, the activation mapping showed that the earliest ventricular activation recorded in left endocardial summit preceding the QRS onset by 28 ms. **e.** Pacing the site with the earliest ventricular activation led a poor pace match to spontaneous PVCs,coincidence just in 6 leads of 12 leads. **f.** Fluoroscopy of the target site by antegrade transseptal approach: The ablation catheter tip was located in the left ventricular endocardial summit, and irrigated RF current was delivered with a target temperature of 43 °C, maximum power output of 35 W, and flow rate of 15 ml/min. The PVCs disappeared after energy delivery for 12.5 s; additional RF current then was applied for another 60s. There was no recurrence during 0.5 years follow-up. Annotation: ABL2 = Ablation catheter in DCGV; ABL1 = Ablation catheter in *left* ventricular endocardial summit; RFon = RFCA begin

Applying the PdW to predict trans-DGCV approach presented with the sensitivity, specificity, positive predictive value, negative predictive value of 100, 78, 61, 100%, applying the IDT to predict trans-DGCV approach presented with the sensitivity, specificity, positive predictive value, negative predictive value of 100, 78, 63, 100%, applying the MDI to predict trans-DGCV approach presented with the sensitivity, specificity, positive predictive value, negative predictive value of 100, 65, 50, 100%. Receiver Operating characterisitcs (ROC) curve showed that PdW, IDT, MDI of VAs had a good ability to differentiate trans-DGCV approach and non-trans-DGCV approach (transaortic and transseptal approach) with area under curve (AUC) of 0.9098, 0.8988, 0.8806 respectively, PdW > 53 ms, IDT > 74 ms, MDI > 0.45 strongly indicated the trans-DGCV approach could firstly be applied for ablation of VAs arising from LV summit, see Fig. 1. The typical electrocardiographic characteristics of VAs from each group were shown in Fig. 2, and a typical case of RFCA procedure from each group was presented in Figs. 3, 4 and 5.

## Discussion

IVAs, including PVCs and VTs, are the most common arrhythmias and usually occur on patients without structural heart disease [1–6]. Although most of them can be safely and efficaciously treated with RFCA, VAs arising from left ventricular summit, which occupies the most superior LV portion, remain a challenge for RFCA^7.^

### Anatomy of the LV summit

Epicardially, the LV summit is the area on the epicardium of the LV beneath the bifurcation of the left main coronary artery and superior to the horizon line of the first septal perforating branch, bounded by the left anterior descending artery (LAD) anteriorly and the left circumflex artery (LCX) laterally, the DGCV transversely bisects the LV summit into a superior portion, which is in close proximity to the proximal coronary arteries with epicardial fat overlaying it and inaccessible to catheter ablation, and an inferior portion that can be accessible by trans-DGCV or transpericardial approach [9]. Endocardially, it is an area within LVOT just below LCC, located anterosuperior of the AMC, and is accessible to endocardial catheter ablation [10].

### The approaches for RFCA of VAs arising from LV summit

Previous studies described different ablation approaches for VAs arising from left ventricular summit. Yamada et al. reported that VAs origin in left ventricular endocardial summit just below LCC can be ablated by a retrograde transaortic approach with a catheter inversion technique [8], meanwhile, he also demonstrated that VA origin in the accessible area of left ventricular epicardial summit can be ablated by a trans-DGCV approach or a pericardial approach via suboxiphoid puncture [9]. Ouyang et al. recently reported that the left ventricular endocardial summit VAs can be better mapped and ablated via a transseptal approach with the ablation catheter reversed S curved [10]. Nonetheless, no matter which ablation approach mentioned above was chosen for left ventricular summit VAs ablation, its advantages and limitations should be taken into full consideration. The transaortic approach is usually firstly attempted for its relatively safe for there is no coronary artery endocardially, however, because of the relatively rigid aortic valve leaflets limiting catheter manipulation and vigorous movements of the aortic valves rendering a contact of the mapping catheter on the tissue in this region unstable, the area below the ASC is sometime insufficiently mapped, leading to unsuccessful ablation. Trans-DGCV approach should be attempted when an epicardial origin within DGCV was supposed, however, it is sometime difficult to place the tip of an ablation catheter in the narrow vascular lumen, meanwhile, high impedance produced during ablation limits the release of energy, and potentially fatal risks, like pericardial tamponade and coronary artery injury, may occur. Transseptal approach was recently proposed for left ventricular summit VAs ablation, by which access into the left ventricular endocardial summit below the ASCs and catheter manipulation in this area are thought easier, however, the transseptal procedure with a Brockenbrough needle is not always safe, catastrophic complications, including atrial rupture and acute mitral valve damage, may be induced by this approach. In addition, Yamada et al. demonstrated by catheter inversion technique via the retrograde transaortic approach, the looped mapping catheter looked the same as the distal half part of the reverse S curve with the antegrade transseptal approach [8], thus they thought inversion with the retrograde transaortic approach should be attempted before the transseptal approach. Pericardial approach via subxiphoid puncture was rarely adopted for its high risk of severe complications, such as heart rupture and pericardial tamponade, and it was forbidden in inaccessible area where lay the proximal coronary arteries and energy application can lead to fatal myocardial infarction. Thus, when VAs were suspected of a LV summit origin, taking full consideration about the ablation approach prior to the procedure was important, it may save the RFCA time and avoid serious complications.

### ECG characteristics of LV summit ECG

Previous studies has described electrocardiographic characteristics criteria to identify the left ventricular summit VAs. Because the LV summit is located most superior in the LV beneath the LCC, VAs originating from this region exhibit high inferior lead R-wave amplitudes and no S wave in V5/V6. Left ventricular summit VAs of left bundle branch block morphology are a rare phenomenon. In our study, all VAs, no matter ablated endocardially and epicardially, had high inferior lead R-wave amplitudes, no S wave in V5/V6, most VAs had right bundle branch block pattern, which is consistent with previous reports [9].

### PdW, IDT, MDI in approach selection

Although a series of ECG characteristics of LV summit VAs have been concluded to differentiate itself from the other sites of PVCs/IVTs origin, the ECG characteristics predicting which ablation approach adopted for LV summit VAs have not been studied. A series of electrocardiographic criteria were once put forward to help recognizing the epicardial origin of VAs and consistently suggested that epicardial VAs usually show prolonged PdW, IDT, MDI [1, 9]. The potential explanation for this electrocardiographic phenomenon is that the Purkinje network is primarily located in the subendocardium, thus excitation of an epicardial ectopy exactly needs more time to reach the Purkinje network to fire arrhythmias than that of an endocardial ectopy, resulting in a relative prolonged PdW, IDT, MDI in epicardial VAs than endocardial VAs. In the present study, we also found that for LV summit VAs, the prolonged PdW time and IDT time and MDI indicated an epicardial origin and trans-DGCV approach could be adopted firstly.

### Catheter ablation of LV summit VAs by transseptal approach

Ouyang et al. described that LV summit VAs ablated by transseptal approach [10], for these VAs, earliest ventricular activation of target sites preceding the QRS onset by 39.5 ± 7.7 ms, pace mapping at the target site showed mismatch of QRS morphology to clinical VAs, radiofrequency resulted in rapid disappearance of VAs within 10 s. We also found the LV summit VAs ablated by transseptal approach showed poor pace match, however, opposed to Ouyang et al., we found the earliest ventricular activation of target sites preceding the QRS onset just by 28.61 ± 2.71 ms, and these VAs usually need more than 10 s’ energy discharging (less than 20 s). The relative delayed ventricular activation time and prolonged energy application, and the poor pace match of target sites by transseptal approach from endocardium, to some extent, suggested that the origin of VAs may be located subepicardially or intramurally. However, complete epicardial mapping via suboxiphoid approach was not performed in any of our patients, whether a much earlier local ventricular activation can be recorded epicardially is unknown.

### Study limitations

In our study, complete mapping of LV summit before ablation by transaortic approach, by trans-DGCV approach, by transseptal approach and by pericardial approach respectively was not performed. The RFCA procedure in the present study was proceeded by transaortic approach and trans-DGCV approach firstly, followed by a transseptal approach. Pericardial approach was abandoned for its high risk of catastrophic complications. Thus where the exactly earliest local ventricular activation of PVCs/IVTs located was not fully investigated. However, for mapping in LV summit VAs, it is something different. The LV summit is the most common area where intramural VAs are located [12]. In the area, even complete mapping from both the endocardium and epicardium performed before ablation, we can not precisely locate the origins of VAs. In our study, as we were more concerned about safety and effectivity of RFCA procedure of LV summit VAs, we performed complete mapping and ablation by transaortic approach and trans-DGCV approach firstly, when failed, the more aggressive transseptal approach was proceeded, by our ablation strategy, a part patients can get away from severe RFCA complications.

## Conclusion

Ventricular arrhythmias arising from the left ventricular summit can be safely eliminated by radiofrequency catheter ablation and many ablation approach are available, electrocardiographic characteristics, PDW >53 ms, IDT > 74 ms, MDI > 0.45 strongly indicated a trans-DGCV approach.

## Abbreviation

AIVV: Anterior intraventricular vein
AMC: Aortic-mitral continuity
ANOVA: Analysis of variance
ASCs: Aortic sinus cusps
CS: Coronary sinus
DGCV: Distal great cardiac vein
ECG: Electrocardiogram
IDT: Intrinsicoid deflection time
IVAs: Idiopathic ventricular arrhythmias
LAD: Left anterior descending artery
LCC: Left coronary cusp
LCX: Left circumflex artery
LV: Left ventricle
LVOT: Left ventricle outflow tract
MDI: Maximal deflection index
NPV: Negative predictive value
PDW: Pseudo delta waves
PPV: Positive predictive value
PVCs: Premature ventricular contractions
RFCA: Radiofrequency catheter ablation
ROC: Receiver operating characteristic
VAs: Ventricular arrhythmias
VTs: Ventricular tachycardias
